# Life Expectancy and Causes of Premature Death by Subgroup for Community-Based Action in Marin County, California, 2017-2021

**DOI:** 10.7759/cureus.51300

**Published:** 2023-12-29

**Authors:** Jasmine Soriano, Lee Ann Prebil, Haylea Hannah, Pooja Mhatre, Lisa Santora, Matthew Willis

**Affiliations:** 1 Epidemiology and Public Health, Marin County Department of Health & Human Services, San Rafael, USA

**Keywords:** small area analysis, premature death, subpopulation health, racial disparities, health equity, life expectancy

## Abstract

Introduction: Marin is a medium-sized county in California’s San Francisco Bay Area. Despite its historically higher-than-average life expectancy and socioeconomic level, known economic and health disparities by race, ethnicity, and geography became more visible during the COVID-19 pandemic.

Methods: We calculated life expectancy, measured years of potential life lost (YPLLs), and described premature mortality for the five years of 2017-2021 by race, ethnicity, census tract, and resource level (as measured by Healthy Places Index [HPI]) to provide data on inequities to guide community-centered action to reduce premature mortality.

Results: Life expectancy for the county was 85.2 years. The non-Hispanic African American/Black population experienced the lowest life expectancy of 77.1 years, 11.6 years lower than the non-Hispanic Asian population which had the highest life expectancy (88.7 years). There was a 14.9-year difference in life expectancy between the census tracts with the lowest (77.1 years) and highest (92.0 years) estimates. We found a moderate, positive association between census tract resource level (HPI) and life expectancy (r=0.58, p<0.01). The leading causes of premature death were cancer, diseases of the circulatory system, and accidental overdoses, with variation by subgroup.

Conclusion: These data highlight health disparities that persist in Marin County and can inform data-driven public health strategies to narrow gaps in longevity between communities.

## Introduction

Marin County in the San Francisco Bay Area has a population of approximately 260,000 residents. Marin has been named the healthiest county in California by the Robert Wood Johnson Foundation’s County Health Rankings in 13 of the 14 years the rankings have been compiled [1]. Despite its high average income level and health status, there are prominent socioeconomic disparities by race, ethnicity, and geography [2]. The County of Marin is committed to advancing equity through dismantling systems that foster inequities and implementing data-driven, targeted interventions to benefit historically marginalized communities.

Life expectancy at birth and years of potential life lost (YPLLs) are indicators of the mortality level and overall health of the population [3]. Identifying leading causes of premature mortality can reveal underlying contributors to health inequities, including those in life expectancy and YPLLs [4]. Identifying the diseases driving lower life expectancy provides insight into the factors that foster inequities [5] and the development of strategies to reverse them. The relationships between life expectancy and race and ethnicity, and life expectancy and resource level, have been well established. Research indicates lower life expectancy for minority racial and ethnic groups compared to the non-Hispanic white population [1, 6-8], and a positive association between life expectancy and resource level [9-13]. There is evidence that disparities in life expectancy by race and ethnicity were exacerbated during the COVID-19 pandemic years in California [6]. 

Producing epidemiological data for subpopulations can highlight disparities among residents and allow local health jurisdictions and their partners, as well as community members themselves, to design programs tailored to the specific needs of their communities. While small-area life expectancy estimates have been made available nationally [14], we aimed to build upon these data with the most current, locally informed, and locally actionable findings. The objectives of this study were to calculate life expectancy at birth, YPLLs, premature mortality rates (PMRs), and describe premature mortality by race, ethnicity, census tract, and resource level. This study employed methods focused on the practical utility of the data. For example, in order to isolate subpopulations with programmatic relevance, we included only community-dwelling residents in census tract analyses by excluding congregate living residents. To explore drivers of disparities in life expectancy by race and ethnicity, we explored cause-specific, age-stratified mortality rates by racial and ethnic group. These research strategies could be translated to other regions and for investigation of other health outcomes.

## Materials and methods

Informed consent

As public health surveillance activities in which human subjects were not involved, this research is exempt from requirements for informed consent under 45 CFR part 46 [15].

Data sources

We used the California Department of Public Health Center for Health Statistics death certificate data from 2017 through 2021. Estimates of the total community-dwelling population of Marin County and its individual census tracts, as well as the population living in group quarters for each census tract, were obtained from the American Community Survey (ACS) five-year estimates for 2015-2019 [16]. California Department of Finance population estimates were used as the source of data on the size of the population in each race and ethnicity. Marin County is home to a state penitentiary, and inmate census data from the San Quentin State Prison were obtained through the California Department of Corrections and Rehabilitation to allow the removal of this population from the estimates. The California Healthy Places Index (HPI) of the Public Health Alliance of Southern California is a measure including 25 socioeconomic characteristics and has been an effective tool in evaluating the impact of social determinants of health [17]. We accessed HPI scores for each census tract in Marin County from the Public Health Alliance of Southern California and organized the tracts into quartiles based on these scores. 

Data analysis

Average life expectancy at birth was calculated for the period 2017-2021 for the county overall, as well as by sex, race/ethnicity, census tract, and HPI quartile. The County Health Rankings and Roadmaps’ Life Expectancy Calculator was used to calculate the five-year average life expectancy at birth and their associated margins of error [1]. Causes of premature mortality, defined as deaths among decedents younger than 75 years of age, were determined by grouping ICD-10-CM codes of primary causes of death using categories defined by the Centers for Disease Control and Prevention Wide-ranging Online Data for Epidemiologic Research [18]. The broad category “External causes of morbidity and mortality” was further separated into “Accidental overdoses,” “Falls,” “Suicides,” “Transport accidents,” and “Other external causes.” YPLLs were estimated by subtracting the age at death from 75 years for all decedents younger than 75 years of age. Rates of YPLLs per 100,000 residents per year were calculated for each population and cause of premature mortality. PMRs, the number of deaths younger than 75 per 100,000 residents age-adjusted to the 2000 standard U.S. population, were calculated for each subgroup. To further examine disparities in causes of death, we calculated cause-specific age-adjusted PMRs by race/ethnicity. 

For analyses of life expectancy, YPLLs, and causes of premature death by census tract, census tracts with fewer than 2,000 residents were combined with neighboring census tracts to produce reliable estimates. The linear regression of HPI score and life expectancy included all census tracts with 2,000 residents or greater. To account for the presence of congregate living facilities - which have a unique mortality experience, are unequally distributed geographically, and include people who reside in that census tract only temporarily - census tract-level analyses excluded deaths in which there were five or greater deaths at a single address from 2017 through 2021, and reduced census tract populations by the number of congregate living residents as determined by the American Community Survey. The threshold of five deaths at a single address optimized the sensitivity and specificity of identifying deaths among congregate living residents. Congregate living settings include skilled nursing facilities and other long-term care facilities, treatment centers, jails, and colleges. Estimates of congregate living residents in each census tract were distributed in age from 20 years and older. We compared census tract-level life expectancy including and excluding congregate living residents (life expectancy and community-based life expectancy). 

State penitentiary inmates were identified in death data by using the occupation and home address fields, both of which contain data identifying decedents as inmates. Inmates were excluded from the numerator and denominator of all analyses. All data were analyzed with Stata/SE 16.1 (Stata Statistical Software, StataCorp., College Station, TX) [19]. Community-based life expectancy by census tract was displayed using an ArcGIS Pro map (Esri., Inc., Redlands, CA) [20]. Other figures were produced using the R package ggplot2 (R Foundation for Statistical Computing, Vienna, Austria) [21-22].

## Results

From 2017 through 2021, there were 10,027 Marin County resident deaths. Average life expectancy at birth for the countywide population was 85.2 years (95% CI: 85.1, 85.4 years) (Table 1). We calculated an age-adjusted PMR of 167.8 deaths per 100,000 residents per year and a YPLL rate of 3,406.5 YPLLs per 100,000 residents per year. Leading causes of premature death were cancer, diseases of the circulatory system, and accidental overdoses (Table 2). 

**Table 1:** Life expectancy, leading causes of premature death, age-adjusted premature mortality rate per 100,000 per year, years of potential life lost (YPLLs) per 100,000 per year: countywide, by race/ethnicity, by sex, and by HPI quartile. State penitentiary inmates were excluded from all analyses.

Population	Population Size	Life Expectancy	Age-Adjusted Premature Mortality per 100,000 per Year	YPLLs per 100,000 per Year	Cause of Premature Death #1 (n)	Cause of Premature Death #2 (n)	Cause of Premature Death #3 (n)
All	255,682	85.2 (85.1, 85.4)	167.77	3,406.47	Cancer (1,040)	Diseases of the circulatory system (595)	Overdoses (188)
Sex							
Female	130,156	87.1 (86.9, 87.4)	123.88	2,402.19	Cancer (527)	Diseases of the circulatory system (183)	Diseases of the nervous system (84)
Male	125,526	83.4 (83.1, 83.6)	218.63	4,391.63	Cancer (513)	Diseases of the circulatory system (412)	Overdoses (141)
Race/Ethnicity							
African American/Black	5,994	77.1 (76.9, 77.3)	463.92	7,802.28	Diseases of the circulatory system (35)	Cancer (25)	Diseases of the respiratory system (13)
Asian	15,372	88.7 (88.6, 88.9)	118.68	2,574.03	Cancer (44)	Diseases of the circulatory system (28)	Diseases of the respiratory system (6)
Hispanic	43,970	87.1 (86.9, 87.3)	147.7	3,130.71	Cancer (73)	Diseases of the circulatory system (34)	Diseases of the digestive system (28)
White	181,806	85.2 (85.0, 85.4)	168.01	3,371.19	Cancer (876)	Diseases of the circulatory system (477)	Overdoses (153)
Other	8,540	85.9 (85.5, 86.2)	168.43	3,224.51	Cancer (16)	Diseases of the circulatory system (14)	Overdoses (6)
Healthy Places Index (HPI) Quartile							
1 (Low)	71,934	82.2 (82.0, 82.4)	224.74	4,177.72	Cancer (274)	Diseases of the circulatory system (201)	Diseases of the respiratory system (74)
2	58,317	84.8 (84.6, 85.0)	158.54	3,164.09	Cancer (245)	Diseases of the circulatory system (141)	Overdoses (45)
3	58,004	85.6 (85.4, 85.8)	151.61	3,058.41	Cancer (244)	Diseases of the circulatory system (108)	Diseases of the digestive system (41)
4 (High)	67,805	86.8 (86.6, 87.0)	133.24	2,608.66	Cancer (258)	Diseases of the circulatory system (113)	Suicides (44)

**Table 2:** Leading 10 causes of premature death, total deaths, age-adjusted premature mortality rates by race and ethnicity, years of potential life lost (YPLLs) per 100,000 residents per year, and median age at death among decedents younger than 75 years of age. State penitentiary inmates were excluded.

Premature Cause of Death (<75)	Total Premature Deaths	Age-Adjusted Premature Mortality Rate (Deaths per 100,000 per Year)						YPLLs per 100,000 per Year	Median Age at Death (<75)
		All	African American /Black	Asian	Hispanic	White	Other		
Cancer	1,040	49.04	83.42	38.35	40.2	49.46	44.57	867.37	67
Diseases of the circulatory system	595	28.21	120.05	24.79	21.45	27.96	37.93	482.79	67
Accidental overdoses	188	15.19	39.63	3.04	5.63	19.54	19.97	400.78	50
Diseases of the respiratory system	180	7.97	45.6	6.06	5.1	8.04	9.98	122.61	69
Diseases of the digestive system	170	9.42	13.12	1.64	13.45	8.49	5.23	200.12	62
Diseases of the nervous system	167	8.09	20.47	5.03	5.34	8.69	0	133.54	69
Suicides	161	12.7	9.99	5.85	9.53	14.32	14.44	343.58	50
Endocrine, nutritional and metabolic diseases	124	6.12	16.99	5.1	5	6	4.7	118.2	65
Mental and behavioral disorders	91	5.2	13.71	2.44	5.39	5.36	6.48	108.66	63
External causes of morbidity and mortality	75	5.06	6.04	4.08	6.73	4.47	1.65	123	59

**Figure 1:**
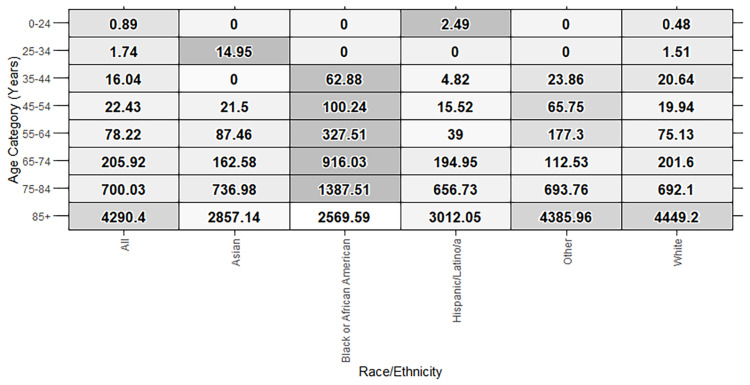
Deaths due to diseases of the circulatory system per 100,000 residents per year by age. State penitentiary inmates were excluded. Cells that are shaded darker represent those with higher mortality rates for each age category (by row).

There was an 11.6-year difference in life expectancy by racial/ethnic population. Non-Hispanic African American/Black life expectancy was lowest at 77.1 years (95% CI: 76.9, 77.3 years) and non-Hispanic Asian life expectancy was highest at 88.7 years (95% CI: 88.6, 88.9 years). The non-Hispanic African American/Black population also had more than double the rate of YPLLs per 100,000 population compared to any other race/ethnicity population. The leading causes of premature death varied by race and ethnicity. While cancer was the leading cause of premature mortality in most racial and ethnic groups, the leading cause in the non-Hispanic African American/Black population was diseases of the circulatory system (ICD-10-CM I00-I99) (Table 1). Further exploration of cause-specific PMRs showed that the non-Hispanic African American/Black population experienced higher rates of premature mortality due to diseases of the circulatory system compared to other racial and ethnic groups in age groups from 35 to 84 years (Figure 1).

**Figure 2:**
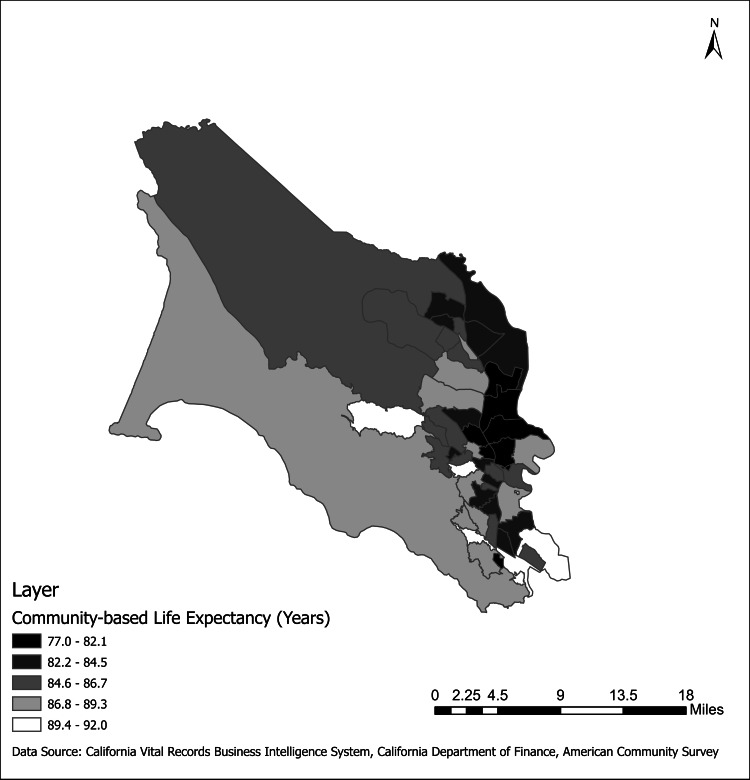
Map of community-based life expectancy by census tract. Census tracts with fewer than 2,000 residents were combined with neighboring census tracts. Congregate living residents (including state penitentiary inmates) were excluded. Areas that are shaded darker have lower community-based life expectancy estimates.

We analyzed the mortality experience of 51 Marin County individuals and combined census tracts (Figure 2). Calculations of community-based life expectancy by census tract revealed a 14.9-year difference between the census tract with the lowest life expectancy (Marin City 77.1 years (95% CI: 75.4, 78.9) and the census tract with the highest life expectancy (Sausalito: Waldo Point 92.0 years (95% CI: 90.3, 93.8). The three leading causes of premature death included cancer in 98.0% of census tracts, diseases of the circulatory system for 100.0% of census tracts, and accidental overdoses in 25.5% of census tracts. Diseases of the digestive system, diseases of the respiratory system, diseases of the nervous system, and suicides were among the three leading causes in 15.7%, 15.7%, 13.7%, and 19.6% of census tracts, respectively.

**Figure 3:**
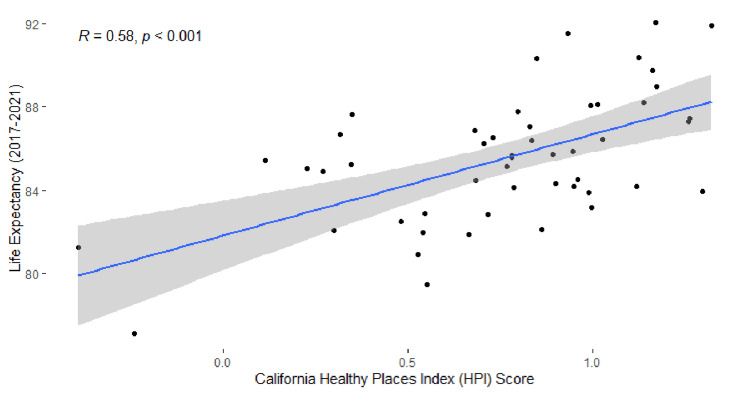
Scatterplot of HPI score and community-based life expectancy by census tract. All census tracts with 2,000 or greater residents were included. Congregate living residents (including state penitentiary inmates) were excluded. The blue line represents predicted results from a fitted linear regression and shaded areas represent 95% confidence intervals.

We found a positive relationship between HPI and life expectancy. Residents of the highest HPI quartile (HPI scores: 1.00 to 1.32) also had the highest life expectancy (86.8 (95% CI: 86.6, 87.0) years), and residents of the lowest HPI quartile (HPI scores: -0.39 to 0.55) had the lowest life expectancy (82.2 years (95% CI: 82.0, 82.4) (Table 1). The ecological correlation between tract-level HPI score and life expectancy was moderate and positive (r = 0.58, p < 0.01) (Figure 3). When congregate living residents were excluded, life expectancy in 43.1% of Marin census tracts or groups of census tracts decreased and 29.4% increased. It remained the same in the other 27.5% of census tracts.

## Discussion

Marin County's life expectancy from 2017 through 2021 was 85.2 years (Table 1). The estimate represents an increase of 1.9 years from internally generated estimates for the period 2005 through 2010 [unpublished data] and is 4.2 and 8.8 years higher than state and national average life expectancy estimates for 2020 and 2021, respectively [23-24]. 

As a local public health department dedicated to supporting the health and well-being of all residents, it is important for Marin County Department of Health and Human Services (Marin HHS) to examine life expectancy and other health outcomes in subpopulations of residents to identify disparities and provide information for targeted intervention. The current data highlight health disparities that persist in Marin by race and ethnicity. Consistent with research among different U.S. populations, we saw a lower life expectancy among the non-Hispanic African American/Black population compared with other racial and ethnic groups [1, 6-8]. Our findings revealed higher mortality from diseases of the circulatory system in the non-Hispanic African American/Black population than other racial and ethnic groups for residents 35 to 84 years of age (Figure 1). This pattern has been seen nationally, with evidence that disparities in cardiovascular disease mortality were exacerbated during the COVID-19 pandemic [25], including a greater increase in cardiovascular mortality among the African American/Black population during the pandemic years compared to other race groups [25-26]. 

Advocates call for a focus on structural changes to racist systems including housing, education, employment, health care, and others to reduce health disparities [13, 27]. Marin County has implemented the Department of Health and Human Services Strategic Plan to Achieve Health and Wellness Equity and Marin County Racial Equity Action Plan to improve the health of historically underserved minorities through system approaches. These estimates of the mortality experience in sub-populations support the goals of existing programs and will continue to shape the development of new efforts. The data will be used by the health department and our partners to make these disparities visible so they can be addressed through tailored outreach. For example, expanding access to high-quality healthcare, a core driver of cardiovascular health [25-27], will be a continued focus in the African American/Black community. This includes targeted efforts to expand access to primary care and improve early detection, disease management, and crisis care.

We also saw differences in community-based life expectancy by census tract with a 14.9-year gap between census tracts with the highest and lowest life expectancy (Table 1, Figure 2). We shared geographic findings publicly through an ArcGIS interactive map displaying community-based life expectancy by census tract. Exploring these neighborhood differences has allowed Marin HHS, community-based organizations, and residents to incorporate findings into the enhancement and development of programs. It is important to acknowledge that, while demographic characteristics and location of residence are not modifiable, causes of premature death are often driven by upstream, modifiable health factors [28]. The significant association between HPI score and life expectancy by census tract is consistent with previous findings and emphasizes the importance of socioeconomic level to community health [17] (Figure 3). To address social determinants of health in addition to direct contributors, Marin HHS values partnerships with community organizations and members. From experiences of COVID-19 response, Marin HHS has formalized these relationships through regional community response teams [29]. Additional efforts will include strengthening collaborations with partners from the communities we are serving to confront the specific geographic patterns through targeted interventions. 

There were limitations to this study. Ecological analyses are susceptible to the “ecological fallacy,” the assumption that characteristics of a population are also true for the individuals within the population. To address this potential effect, we share results alongside explanations that average life expectancy and its relationship with other factors provide valuable information about the group, but not any individual member of the group. Additionally, life expectancy does not always reflect the mortality level of a population [30]. There are several potential effects due to short-term population fluctuations that could limit our interpretation of life expectancy calculations. Our approach of including the five-year period could have reduced the likelihood of fluctuation effects that influence life expectancy estimates using shorter timeframes. However, the timeframe of 2017 through 2021 did not allow for an examination of the impact of the COVID-19 pandemic. Life expectancy was projected to continue to increase nationally by 2040 [28], however, recent data reveal that national life expectancy declined in 2021 for the second year in a row, driven by increases in COVID-19, unintentional injury (including overdoses), heart disease, liver disease, and suicide [8, 24]. Continued analyses are needed to monitor the impact of the COVID-19 pandemic on local life expectancy.

Another challenge in subpopulation analyses is that, even including a five-year period, some groups of interest were too small to produce reliable life expectancy estimates. This required combining census tracts with fewer than 2,000 residents with neighboring census tracts for most analyses and including several race groups into a “Non-Hispanic Other” category. While there is evidence that the American Indian/Alaska Native population has experienced lower life expectancy than other race groups in other U.S. populations [6-7, 24], the small population in Marin County prevented the investigation of this group alone. The method of combining small populations is a demonstration of how other jurisdictions could address small populations.

Lastly, we recognize that population estimates can be flawed and, to the extent that they are under or overestimates for any particular group or subgroup, life expectancy, YPLL, and PMR data will be incorrect. Estimates are unlikely to fully account for migration during the COVID-19 pandemic. Even though the particular findings may be subject to error, communicating the data with these limitations has been a helpful tool to drive our local work. 

Despite these limitations, this study demonstrates how local health jurisdictions can effectively use hyper-local mortality data to inform action. We designed the analyses to optimize the usefulness of the data to County and community-based interventions. We took several steps that improved the relevance of the findings: calculating life expectancy for demographic subgroups and sub-county areas, describing causes of premature death and cause-specific mortality rates to better understand the drivers of lower life expectancy, excluding inmate populations, and excluding congregate living residents in census tract analyses for a more accurate depiction of mortality among community-dwelling residents. These aspects of the research, coupled with the dissemination of findings in diverse formats and venues, illustrate how local applied epidemiology can be incorporated into equity-advancing initiatives. Marin HHS will continue to use these local, actionable data to conduct localized public health. Additionally, sharing methods with other jurisdictions could allow for mutual learning in producing small-area life expectancy and mortality data. 

## Conclusions

Our findings provide a demonstration of how subgroup analysis of life expectancy and premature mortality provides an important tool for public health programming, even in a county with high overall life expectancy. We found inequities in life expectancy by race, ethnicity, neighborhood, and socioeconomic level that can be addressed in order to improve the health outcomes of residents who need it most. These data will continue to be used to shape our priorities, partnerships, and programs.
